# FACE-ing the future of single-pixel complex-field microscopy beyond the visible spectrum

**DOI:** 10.1038/s41377-025-02077-5

**Published:** 2026-01-01

**Authors:** Stefan G. Stanciu, Edoardo Charbon

**Affiliations:** 1 Photon-X Spectrum Lab, CAMPUS Research Institute, National University of Science and Technology POLITEHNICA Bucharest, Bucharest, Romania; 2 Center for Microscopy-Microanalysis and Information Processing, National University of Science and Technology POLITEHNICA Bucharest, Bucharest, Romania; 3 Advanced Quantum Architecture Lab (AQUA), Swiss Federal Institute of Technology in Lausanne (EPFL), Lausanne, Switzerland; 4 Center for Quantum Science and Engineering, EPFL, Lausanne, Switzerland

**Keywords:** Imaging and sensing, Biophotonics

## Abstract

Single-pixel imaging (SPI) has long been recognized for its potential in spectral regions where conventional imaging sensors fall short, such as the near-infrared spectrum. Yet, despite its sensitivity, SPI and its complex-field variants have faced critical bottlenecks in speed and throughput, hindering their adoption for real-time applications. A recently proposed approach—frequency-comb acousto-optic coherent encoding (FACE)—places an important step in overcoming these barriers, delivering an unprecedented space-bandwidth-time product. By showcasing its versatility through several compelling proof-of-concept demonstrations in real-time complex-field microscopy, this advance paves the way for transformative progress in optical imaging beyond the visible spectrum. We discuss here advantages, challenges and potential future directions for scaling up this technology.

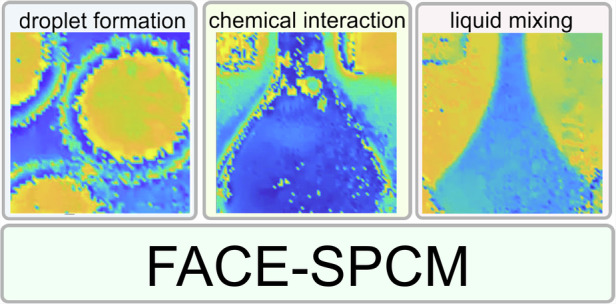

## Abstract (web summary)

Examples on the utility of single-pixel complex-field microscopy with frequency-comb acousto-optic coherent encoding (FACE-SPCM) as demonstrated by Wu et al., by phase imaging inside a microfluidic device. Left: Droplet formation with water and oil as dispersed and carrier medium, respectively. Center: Chemical reaction between acetic acid and baking soda. Right: non-reactive liquid mixing of ethanol and pure water. FACE-SPCM images adapted from Wu et al.^1^

## Complex-field imaging in challenging spectral regimes

The 21st century has been characterized by significant advancements in optical imaging technologies operating within the visible spectrum, propelled by the maturity level of silicon-based detectors and the wide variety of advanced image sensors. In contrast, extending imaging capabilities beyond the visible—into the near-infrared (NIR), terahertz, or X-ray regions—remains both technically and economically challenging, largely due to the limited availability of high-performance pixel-array detectors in these spectral domains, and intrinsically high costs. This limitation poses a significant barrier to scientific progress, as non-visible wavelengths can reveal critical information about biological structures, such as proteins, cells, and tissues^2^, advanced materials, and chemical processes—insights that are often beyond the reach of visible-light imaging. For example, NIR light offers label-free contrast by penetrating deeper into tissue^3,4^ and interacting with endogenous chromophores^5^, which is a fundamental advantage, especially for in-vivo imaging^6,7^. Furthermore, label-free bioimaging^5^, often associated with NIR techniques^2^, is of critical importance for elucidating not only endogenous structures and processes, but also their interactions with exogenous agents, such as theranostic probes.

Single-pixel detectors, in contrast to pixel arrays, maintain high quantum efficiency across a broad spectral range, including the mid-infrared spectrum^8^, and are cost-effective. Building on this foundation, single-pixel imaging (SPI) has evolved into a powerful computational approach for imaging in “sensor-limited” regimes. SPI attracted significant interest, also given some apparent synergies with artificial intelligence^9,10^. SPI systems sequentially project known patterns onto a scene and reconstruct images from a series of intensity measurements captured by a single-element detector^11^. The approach also supports single-pixel complex-field imaging^12^, which retrieves both amplitude and phase—essential for visualizing transparent samples or dynamic refractive index changes. This approach builds on the earlier findings of Clemente et al., who showed how phase imaging can be achieved using a single-pixel detector in a homodyne “ghost” holography setup^13^.

Despite their flexibility, SPI techniques have long been constrained by a low space-bandwidth-time product (SBP-T)—a critical measure of the information volume captured per unit time. This bottleneck arises from the inherently sequential nature of pattern projection and the heavy computational cost of reconstructions, rendering SPI impractical for high-speed dynamic imaging.

## The FACE approach: a leap in throughput

In a recent *Light: Science & Applications* publication, Wu et al.^1^ introduce a novel technique called frequency-comb acousto-optic coherent encoding (FACE), which sets a new benchmark for single-pixel complex-field imaging. Their system delivers real-time, high-resolution, phase-resolved imaging at 1,000 Hz, with an 80 × 81-pixel frame, a lateral resolution of 3.76 μm, and a field of view of about 300 μm—resulting in a record-breaking SBP-T of 1.3 × 10⁷, demonstrated within a Single-Pixel Complex-Field Microscopy (SPCM) platform.

This marks a 2–3 order of magnitude improvement over conventional SPI approaches and even exceeds the information throughput of certain commercial NIR cameras. Notably, this advancement was accomplished without relying on mechanical scanning or high-speed modulators—elements that previously added instability, complexity, and sensitivity loss to previous designs.

## Key principle: frequency-comb encoding without moving parts

**Fig. 1:**
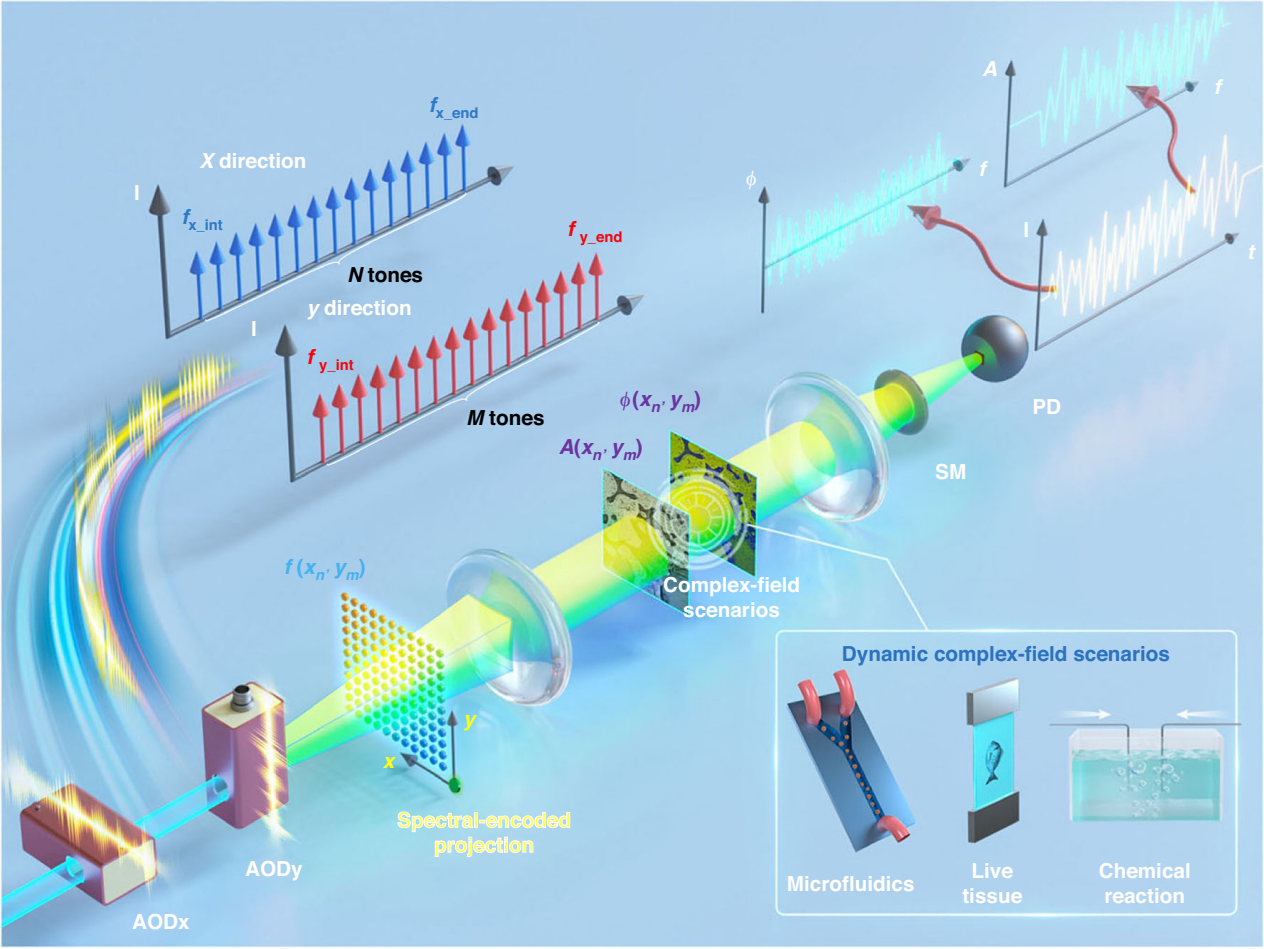
**Schematic illustration of the FACE-SPCM system**. A two-dimensional spectrally encoded projection is created using a pair of orthogonal optical frequency combs modulated via acousto-optic deflectors. The complex-field information of the scene is embedded into a temporal signal through a coaxial heterodyne holographic setup. This configuration allows for efficient recovery of amplitude and phase details in dynamic environments, as demonstrated by the authors, such as microfluidic flows, live biological tissues, and chemical processes. AODx (AODy) acousto-optic deflector along the x (y) axis, SM scattering medium, PD single-pixel photodetector. Figure reproduced from Wu et al.^1^, available under CC-BY license

The FACE system overcomes the speed constraints of conventional spatial light modulators by employing acousto-optic deflectors (AODs) driven by orthogonal frequency-comb signals (Fig. 1). This configuration projects a two-dimensional spatial pattern onto the sample, with each spatial position uniquely encoded by a specific frequency tone. As a result, the system generates a continuously evolving 2D pattern without mechanical movement—a truly motionless approach that enables ultra-fast encoding. A single-pixel photodetector captures the signal, while spatial information is reconstructed via Fourier transform-based decoding, facilitated by a coaxial heterodyne holography setup. A stable zero-order reference beam is maintained within the illumination path, ensuring robust, high-fidelity recovery of both phase and amplitude information.

The strength of FACE lies in its inherent parallelism: rather than projecting patterns sequentially, it encodes and projects all spatial frequencies at once, embedding spatial information in the temporal evolution of the signal. Leveraging digital signal processing for frequency generation and GPU-accelerated reconstruction further boosts its real-time performance.

## Dynamic imaging with FACE-SPCM: from droplets to diffusion

To validate their approach, Wu et al. first assessed system performance on a resolution target, confirming the reported lateral resolution of 3.76 μm. They then showcased the versatility of FACE-SPCM by demonstrating its effectiveness across a range of rapid, dynamic, and phase-sensitive imaging scenarios. Microfluidic imaging revealed detailed dynamics of oil-encapsulated water droplets as they formed and flowed through a chip—processes that conventional bright-field microscopes struggled to capture due to insufficient speed or contrast. FACE-SPCM provided phase-resolved movies at 1 ms intervals, even through scattering media. In a second set of experiments, detailed in one of the Supplementary Notes of the original article, live microorganism tracking demonstrated the ability to visualize and monitor paramecia swimming in aqueous media, highlighting the technique’s relevance for label-free biological imaging. In a third set of experiments, chemical reaction monitoring illustrated the detection of subtle phase differences during an acetic acid–sodium bicarbonate reaction. As the reactants mixed and produced CO₂, FACE-SPCM captured both the chemical interface and the resultant microbubble formation in real time. Using the same microfluidic setup, liquid mixing without chemical reaction was assessed as well, as a control experiment, for ethanol and pure water. These demonstrations collectively emphasize the power of phase contrast and throughput, thereby distinguishing dynamics in otherwise transparent media and revealing structure and motion invisible to intensity-only imaging techniques.

## Implications of FACE for opening up new research avenues

By achieving an SBP-T of 1.3 × 10⁷, the FACE-SPCM system not only outperforms previous SPI/SPCM implementations but also rivals (and in some cases, surpasses) commercial pixel-array cameras in throughput—while maintaining the inherent advantages of SPI: operation across exotic spectra, low detector cost, and system simplicity. Furthermore, the FACE architecture is spectrally flexible. With appropriate AODs, light sources, and detectors, the system could be extended from ultraviolet to mid-infrared regimes—making it a platform technology rather than a niche solution. The ability to perform real-time, phase-sensitive imaging in the NIR opens new doors in in critical fields, as demonstrated by the authors:

- Biomedical imaging, such as monitoring live cells or tissue dynamics label-free.
- Environmental sensing, for flow monitoring in optically challenging conditions.
- Chemical process control, where transparent mixing and reactions can be monitored continuously and non-invasively.

### Expert perspective: strengths, challenges, and future research opportunities for scaling up the FACE technology

In conventional systems, the Digital Micromirror Device (DMD) dictates the speed at which spatial patterns can be projected. Since DMDs rely on mechanical micromirrors that physically tilt, their switching rates are limited—typically to tens of kHz^11^—creating a major bottleneck for high-speed or real-time imaging. FACE eliminates this limitation by replacing DMDs with ultrafast acousto-optic modulation. Second, it removes the need for sequential pattern projection by introducing a parallel frequency-encoding strategy. Third, it supports real-time processing through efficient FFT-based decoding combined with hardware acceleration. In addition, the system’s modularity makes it well-suited for compact or portable implementations, particularly in resource-constrained or field-deployed environments. However, it should be considered that scaling to higher resolutions will depend on the development of GHz-bandwidth acusto-optic deflectors and matching high-speed detectors. But even in its current form, FACE sets a new benchmark and significantly elevates the capabilities of SPI/SPCM platforms.

As a future direction, it would perhaps make sense, without defying the purpose of this work, to consider the use of hybrid approach, where a multi-detector macro-pixel could be used to further enhance speed and potentially resolution, which at 80 $\times$ 81 is now relatively low. In this way, one could relax pitch and fill factor constraints, while improving speed and/or spectral performance of the overall system. Also looking forward, integration with compressive sensing^14^ may further reduce the number of required measurements, allowing larger frame sizes or higher FPS.

Another area for investigation is hyper- or multi-spectral imaging, where maintaining parallelism while managing multiple optical channels may require careful balancing between channel count and speed. Similarly, a systematic study of trade-offs between noise, sensitivity, and speed for different formats, not addressed in the current work, could provide valuable insights into the practical limits of resolution and form factor. To this end, it may also be worth considering in the future to combine FACE-SPCM with super-resolution methods^15,16^, that could offer potential routes to enable the recovery and analysis of fine structural details in the acquired data without altering the original acquisition hardware.

FACE has made significant progress in addressing bandwidth constraints, yet there remain interesting directions to explore with emerging imaging modalities, such as quanta image sensors and single-photon imaging. These photon-starved regimes rely on high levels of parallelism to achieve quantum advantages, for instance via photon locality and coincidence effects. Observing phenomena like the Hanbury Brown and Twiss effect^17^ generally involves detecting single photons at separate locations simultaneously, which presents opportunities for further adaptation of the FACE approach.

Applications in more complex quantum imaging setups, such as those involving multiple or entangled photons, e.g., Hong-Ou-Mandel interference, also present promising avenues for optimization, particularly when combined with spectroscopic or polarimetric analysis.

It is also important to recall that while SPI techniques are naturally suited to static scenes, real-world scenarios often involve motion, which can lead to blurring. This is of course also the case in SPCM investigations on dynamic environments, such as those discussed by the authors. Techniques such as Quanta Burst Photography^18^ offer inspiration for addressing motion effects, and there is potential for FACE to integrate similar approaches, potentially leveraging intelligent algorithms or deep learning to further enhance performance.

Another promising direction for future research lies in developing correlative imaging workflows^19^ that combine FACE-SPCM with other high-resolution modalities, particularly those that are similarly label-free^5^. One intriguing possibility would be pairing FACE-SPCM with scattering-type Scanning Near-Field Optical Microscopy (s-SNOM)—a technique that offers nanoscale access to amplitude, phase, and complex optical constants at wavelengths ranging from visible to THz^20,21^—enabling a multi-scale approach to mapping optical properties across different length scales.

When it comes to resolution, with the rise of label-free optical nanoscopy^5^ the question remains whether FACE-SPCM could one day approach the nanoscale. While this would require major advances beyond its current principles, computational strategies such as image-to-image translation^22^ may provide a practical route for synthetic resolution enhancement in the near term. Nonetheless, the prospect of achieving true nanoscale resolution with FACE-SPCM variants through purely optical or photonic innovations remains a fascinating, albeit highly challenging, area for future exploration.

## Conclusion

Wu et al.^1^ have shown that the long-standing trade-offs among speed, resolution, and field of view in single-pixel microscopy can be redefined. With the FACE-SPCM system, they present a powerful solution for high-throughput, complex-field imaging—one that is fast, versatile, and capable of probing spectral ranges that conventional Si, GaAs, InGaAs, or HgCdTe detectors cannot access, such as the large parts of the infrared. By removing the traditional bottlenecks of SPI and SPCM, the FACE architecture elevates what was once a niche computational technique into a scalable, real-time imaging platform. The current work suggests that the future of label-free, high-speed microscopy—especially in spectral domains where conventional sensors falter—may indeed lie in single-pixel approaches. Still, important limitations remain, leaving ample opportunity for further advances in the field.
